# The Role of Notch, Hedgehog, and Wnt Signaling Pathways in the Resistance of Tumors to Anticancer Therapies

**DOI:** 10.3389/fcell.2021.650772

**Published:** 2021-04-22

**Authors:** Vivek Kumar, Mohit Vashishta, Lin Kong, Xiaodong Wu, Jiade J. Lu, Chandan Guha, B. S. Dwarakanath

**Affiliations:** ^1^R&D Dept, Shanghai Proton and Heavy Ion Center (SPHIC), Shanghai, China; ^2^Shanghai Key Laboratory of Radiation Oncology (20dz2261000), Shanghai, China; ^3^Shanghai Engineering Research Center of Proton and Heavy Ion Radiation Therapy, Shanghai, China; ^4^Department of Radiation Oncology, Shanghai Proton and Heavy Ion Center, Fudan University Cancer Hospital, Shanghai, China; ^5^Albert Einstein College of Medicine, The Bronx, NY, United States

**Keywords:** Notch signaling, Hedgehog signaling, Wnt signaling, tumors, resistance, anticancer, therapy

## Abstract

Resistance to therapy is the major hurdle in the current cancer management. Cancer cells often rewire their cellular process to alternate mechanisms to resist the deleterious effect mounted by different therapeutic approaches. The major signaling pathways involved in the developmental process, such as Notch, Hedgehog, and Wnt, play a vital role in development, tumorigenesis, and also in the resistance to the various anticancer therapies. Understanding how cancer utilizes these developmental pathways in acquiring the resistance to the multi-therapeutic approach cancer can give rise to a new insight of the anti-therapy resistance mechanisms, which can be explored for the development of a novel therapeutic approach. We present a brief overview of Notch, Hedgehog, and Wnt signaling pathways in cancer and its role in providing resistance to various cancer treatment modalities such as chemotherapy, radiotherapy, molecular targeted therapy, and immunotherapy. Understanding the importance of these molecular networks will provide a rational basis for novel and safer combined anticancer therapeutic approaches for the improvement of cancer treatment by overcoming drug resistance.

## Introduction

The emergence of resistance to anticancer therapeutics is one of the major barriers that limit the efficacy of cancer therapy (Vasan et al., 2019). Resistance develops during chemotherapy, radiotherapy, molecularly targeted therapy, and immunotherapy in most cancer patients and prevents long-term survival. Resistance to therapy can be classified as intrinsic and acquired. The intrinsic resistance occurs from the sub-population of cancer cells, which already have the capability to counter a given therapy due to the pre-existing genotypic or phenotypic alterations. In contrast, the acquired resistance develops during the treatment by the therapy-induced selection of pre-existing resistant cellular state or by acquisition/adaptation to new genotype/phenotype changes required to withstand the therapy. In both these cases, major signaling pathways involved in the developmental process plays a vital role (Holohan et al., 2013; Chatterjee and Bivona, 2019).

Notch, Hedgehog, and Wnt form the major developmental signaling pathways which play a fundamental role in the dynamic transformation of a single-celled zygote into a highly complex multicellular organism (Collu et al., 2014; Siebel and Lendahl, 2017). These signaling pathways regulate the core cellular processes, including proliferation, differentiation, and migration, that collectively underlie organismal growth and development. The roles of these signaling pathways are not only restricted to the functioning of terminally differentiated somatic normal cells but also encompass adult stem cell niches, which serve to maintain the functional integrity of tissue and organs (Takebe et al., 2015; Clara et al., 2020). Due to the involvement of these signaling pathways in the basic cellular processes, its dysregulation often leads to diseases state, including cancer. Moreover, it is now widely recognized that aberrant regulation of these developmental signaling pathways plays a crucial role in providing resistance to various anticancer therapy (Takebe et al., 2015). Here, we present an overview of the role of Notch, Hedgehog, and Wnt signaling pathways in providing resistance against contemporary anticancer therapies, including molecular targeted therapy and the emerging immunotherapy. We also briefly present the current status of clinical trials that evaluate drugs targeting these signaling pathways and discuss how they can be explored for the development of novel and safer combinatorial approaches for overcoming drug resistance and enhanced efficacy.

## Major Developmental Signaling in Cancer

### Notch Signaling

The Notch is an evolutionarily conserved signaling pathway involved in many developmental and cellular processes, starting from the germ layer formation to the differentiation of specialized cell types in the embryo (Artavanis-Tsakonas et al., 1999). In the adult, it plays an important role in various cellular processes such as cell-fate specification, differentiation, proliferation, adhesion, apoptosis, migration, angiogenesis, epithelial-mesenchymal transition, and stem cell maintenance (Hori et al., 2013). In mammals, Notch signals through four Notch receptors (Notch 1–4) and five ligands (Jagged-1, -2, and Delta-like-1, -3, and -4), which are all type I transmembrane protein. The Notch receptor consists of an extracellular domain which contains 29–36 epidermal growth factors (EGF)-like repeats (involved in ligand-binding), the transmembrane domain, and an intracellular domain consisting of RAM domain; the Ankyrin repeats, transcriptional activator domain (TAD), and PEST domain (Gordon et al., 2007). The Notch ligands are composed of EGF-like repeats in the extracellular domain, DSL domain, and cysteine-rich region in Serrate.

**FIGURE 1:**
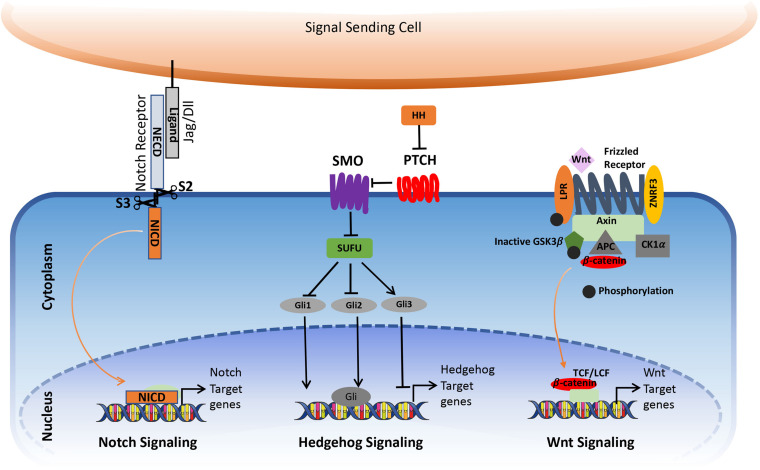
Simplified view of canonical Notch, Hedgehog, and Wnt signaling pathway in cancer. The figure is widely discussed in the text.

Notch mediates short-range intercellular communication through interaction with ligands present on the neighboring cells. The binding of Notch ligands to the Notch receptors triggers S2 cleavage by ADAM10 and ADAM17 in the extracellular part of the receptor, leading to the shedding of the extracellular part. This is followed by the S3 cleavage in the transmembrane portion by γ-secretase. After S3 cleavage, the Intracellular domain (ICN) is released from the plasma membrane, which translocates to the nucleus. Here, it interacts with RBPJ—recombining binding protein suppressor of hairless/CBF1/suppressor of hairless/Lag-1 (RBPJK/CSL) and convert/transform the repressor complex into coactivator complex, thus promoting the transcription of the target genes (Figure 1; Kopan and Ilagan, 2009; Kopan, 2012).

In the recent past, increasing evidences suggest the existence of non-canonical Notch signaling that is independent of canonical Notch ligand or transcription factor CSL/RBPJ (Andersen et al., 2012; Ayaz and Osborne, 2014). In this regard, other ligands that are non-canonical Notch ligands (Jagged/Dll type) have been reported to activate Notch signaling. For example, DLK1 (non-canonical Notch ligand) can directly interact with Notch and control Notch signaling. DlK1 are elevated in ovarian cancer and promotes tumorigenesis and epithelial-mesenchymal transition of high-grade ovarian carcinoma through activation of Notch signaling (Huang C. C. et al., 2019). Similarly, molecules such as Delta/Notch like EGF-related receptor (DNER) (Eiraku et al., 2002) and MB3 and contractin1 (D’Souza et al., 2008) have seen shown to have ligand activity and can activate Notch signaling independent of canonical Notch ligands. Non-canonical Notch signaling can also occur in CSL/RBPJ independent manner, where the activated Notch receptor interacts with various molecules other than CSL/RBPJ to exert its cellular process. This mode of non-canonical Notch signaling has been shown to modulate various signaling pathways such as NfKB, Pi3K, AKT, mTOR, HIF-1a, Wnt, etc., that have an important role in developmental process and cancer. For example, in T-ALL, NICD directly interacts with IKKa to maintain NfKB activity (Vacca et al., 2006). In cervical cancer, Notch activates the PI3K pathway that is independent of CSL (Veeraraghavalu et al., 2005). Moreover, in CSL-deficient mammary gland, some of the Notch mediated response was observed, confirming the existence of non-canonical Notch signaling independent of CSL. In addition, the role of non-canonical Notch signaling has also been reported in DNA damage response (DDR), where Notch1 directly interacts with ATM through FATC domain and inhibits its activation by impairing the formation of ATM-FOXO3a-KAT5/Tip60 Complex. Moreover, the negative correlation between Notch1 and ATM activation has been observed in human breast cancer, and it contributes to the survival of Notch1 driven leukemia cells upon DNA damage (Vermezovic et al., 2015; Adamowicz et al., 2016). Recently, Notch intracellular domain (NICD) was shown to interact with STING at the cyclic dinucleotide (CDN) binding domain, which resulted in inhibition of STING activation. This affected the apoptosis and necroptosis in a variety of immune cells, including T cells (Long et al., 2020). Thus the non-canonical Notch signaling represents an exciting avenue for future research, which may reveal novel strategies to block Notch signaling in diseases and chemoresistance.

As Notch signaling is involved in many crucial cellular functions, its aberrant regulation/expression often leads to pathological events ranging from developmental disorders to cancer. Indeed, many observations suggest that alterations in Notch signaling are associated with many human cancers. Moreover, Notch receptors and ligands have been found as prognostic markers in human cancers. Initially, the oncogenic role of Notch signaling was documented in T cell Acute Lymphoblastic Leukemia (T-ALL), where the activating mutations in NOTCH1 were suggested to be a major mediator for the development of malignancy (Ellisen et al., 1991). Later, its role in other hematological malignancies such as B-cell chronic lymphocytic leukemia (B-CLL) (Rosati et al., 2009), mantle cells lymphoma (MCL), Multiple Myeloma, AML, etc. has been well established (Gragnani et al., 2020). Recently, a genome-wide study in relapse cases of T-cell lymphoblastic lymphoma (T-LBL) identified Notch1 as the putative driver for relapse and malignancy (Khanam et al., 2020). Moreover, activation of Notch signaling by the T cell in the tumor microenvironment was demonstrated as key mediator of Akt-induces RT (Richter’s transformation), which is an aggressive lymphoma that occurs upon progression from chronic lymphocytic leukemia (CLL), suggesting the critical role of Notch signaling in RT transformation (Kohlhaas et al., 2021). While NOTCH receptor mutations are uncommon in other tumor forms, NOTCH is aberrantly activated via various mechanisms in several malignancies, including colorectal and pancreatic cancer, melanoma, medulloblastoma, and adenocystic carcinoma (Ranganathan et al., 2011). Conversely, NOTCH can also function as a tumor suppressor as observed in various solid tumors such as squamous cell carcinoma, liver cancer, small-cell lung cancer, etc., where the loss of function mutations in NOTCH1/2/3 have been identified (Nowell and Radtke, 2017).

### Hedgehog Signaling

Hedgehog signaling is an evolutionarily conserved pathway that regulates the morphogenesis of various organs during embryogenesis and postnatal development. It regulates diverse cellular processes in the adult, including proliferation, tissue differentiation, and repair of normal tissues. In addition, it is also involved in stem cell renewal and organ homeostasis. The major components of the Hedgehog signaling pathways are the Hh ligands Sonic hedgehog (Shh), Indian Hedgehog (IHH) and Desert hedgehog (DHH), Patched (PTCH), which is a 12-transmembrane domain receptor protein, which locates in the primary cilium, the 7-transmembrane G-protein coupled receptor Smoothened (SMO), the suppressor of fused protein (SUFU) in the cytoplasm, and the glioma-associated oncogene (GLI) transcription factors.

In the absence of ligands, Ptch1 inhibits SMO accumulation in primary cilia, thereby resulting in the block of the pathway activity. The binding of the Hedgehog ligands to Ptch1 causes internalization and degradation of the Ptch receptor, thereby releasing SMO to enter the primary cilia where it promotes the dissociation of a SUFU– GLI complex. This activates GLI transcriptional activators and their translocation into the nucleus to activate the expression of Hedgehog target genes such as GLI1 and Ptch (Figure 1). In addition to the canonical activation of the Hedgehog pathway, growing evidence points toward non-canonical mechanisms through which hedgehog signaling gets activated, which can contribute to the development of several types of cancer.

Ectopic activation of the Hedgehog signaling is implicated in several cancers, including hematological malignancies and solid tumors, where it is associated with tumor development, progression, and recurrence after anticancer therapy. In hematological malignancies, the role of Hh signaling in maintaining leukemic stem cells has been well established. Thus, inhibition of HH signaling reduces the stem cell potential to initiate leukemia (Fukushima et al., 2016). This effect was observed in chronic myeloid leukemia (CML), where inhibition of HH signaling reduced the development of leukemia and enhanced the survival of the CML mouse model (Irvine et al., 2016). Similarly, in cells from patients with acute myeloid leukemia (AML), the inhibition of HH signaling in combination with 5-azacytidine showed synergistic efficacy due to reduced stem cell potential to initiate leukemia (Tibes et al., 2015). Moreover, high frequencies of somatic mutations of Ptch1 (70–90%) and a lesser extent in Smoothened (10–20%) are reported in human basal cell carcinoma (Bonilla et al., 2016).

### Wnt Signaling

Wnt signaling pathway is another evolutionally conserved pathway that directs developmental processes, stem cell proliferation, and tissue homeostasis throughout the metazoans (MacDonald et al., 2009). Hence, any perturbation due to physiological stress in the Wnt signaling pathway results in pathological conditions such as birth defects, cancers, etc. (Clevers, 2006). In humans, 19 genes are encoding WNTs that bind to various receptors and stimulate different intracellular signal transduction pathways (Niehrs, 2012). Recent studies on the WNT pathway roughly divided it into either canonical (β-catenin dependent) or non-canonical (β-catenin independent) signaling pathways (Niehrs, 2012). Depending upon their potential to induce morphological transformation in a murine mammary epithelial cell line (C57MG), the Wnt family has been categorized into different types (Wong et al., 1994). Wnt1, Wnt3, Wnt3a, and Wnt7a fall under the category of highly transforming members, and Wnt2, Wnt4, Wnt5a, Wnt5b, Wnt6, Wnt7b, and Wnt11 are grouped under intermediately transforming or non-transforming members (Kikuchi et al., 2011). In general, Frizzled proteins function as common receptors for both canonical as well as non-canonical pathways (Niehrs, 2012).

The canonical Wnt signaling pathway is a well-studied pathway that is activated by the interaction of Wnt with a Frizzled (Fz) receptor and LRP5/LRP6, where LRP stands for lipoprotein receptor-related protein (which is a single-span trans-membrane receptor) (Niehrs, 2012). Followed by the ligation of Wnt and the Fz/LRP co-receptor complex, the canonical signaling pathway gets stimulated. The Fz can interact with a cytoplasmic protein called Disheveled (Dsh), which acts upstream of β-catenin GSK3β (Clevers, 2006). Another cytoplasmic protein, Axin, interacts with the intracellular domain of LRP5/6 through five phosphorylated PPPSP motifs in the cytoplasmic tail of LRP. GSK3 phosphorylates PPPSP motifs, whereas Casein kinase 1-γ (CK-1γ) phosphorylates multiple sites within LRP5/6, which in turn promotes the recruitment of Axin to LRP5/6. CK-1γ isoforms within the CK-1 family carry putative palmitoylation sites at the carboxy-terminal (Figure 1; Davidson et al., 2005).

In the inactivated/un-stimulated state, GSK-3 phosphorylates the transcriptional coactivator β-catenin that renders it in an active state. Inactivation of β-catenin is characterized by the formation of a “destruction complex” that comprises of GSK3, adenomatosis polyposis coli (APC), Axin, and casein kinase Iα (CKIα) (Niehrs, 2012) which leads to the ubiquitination of β-catenin by an E3 ubiquitin ligase called β-TrCP. This complex targets it for proteasomal degradation (Krishnamurthy and Kurzrock, 2018). This results in the absence of β-catenin to the nucleus and the repressor complex containing T-cell specific factor (TCF)/lymphoid enhancer-binding factor (LEF), and transducing-like enhancer protein (TLE)/Groucho binds and represses the activity of the target gene (Levanon et al., 1998; Brantjes et al., 2001; MacDonald et al., 2009). Following the binding of Wnt to Frizzled-Axin-LRP-5/6 complex, cytosolic GSK-3β (Glycogen synthase kinase-3 beta) is sequestered, and the phosphorylation of β-catenin is inhibited. The accumulation of hypo-phosphorylated β-catenin in the cytosol allows its migration to the nucleus, where it regulates target gene expression by interacting with the TCF/LEF family of transcription factors. This signaling is implicated in the regulation of cell differentiation and proliferation (Gordon and Nusse, 2006; Zhan et al., 2017).

Wnt signaling has a prominent role in carcinogenesis and has been widely studied in colorectal cancer. Indeed, the first evidence of dysregulated Wnt signaling came from the studies of hereditary colorectal cancer. It was found that this aberrant activation of Wnt signaling in colorectal cancer was due to the loss of function mutations in the APC gene or point mutation in the N-terminal sites of β-catenin that leads to the stabilization of β-catenin. Later, studies from several groups showed the activating mutation in Wnt signaling components in various cancer such as breast cancer, pancreatic cancer, melanoma, lung cancer, prostate, etc. (Zhang and Wang, 2020). Moreover, Aberrant WNT signaling has been reported in several hematological malignancies such as AML, CML, B-ALL, Multiple Myeloma, CLL, etc. (Frenquelli and Tonon, 2020).

## Crosstalk Between Notch, Hedgehog, and Wnt Pathway in Cancer

**FIGURE 2:**
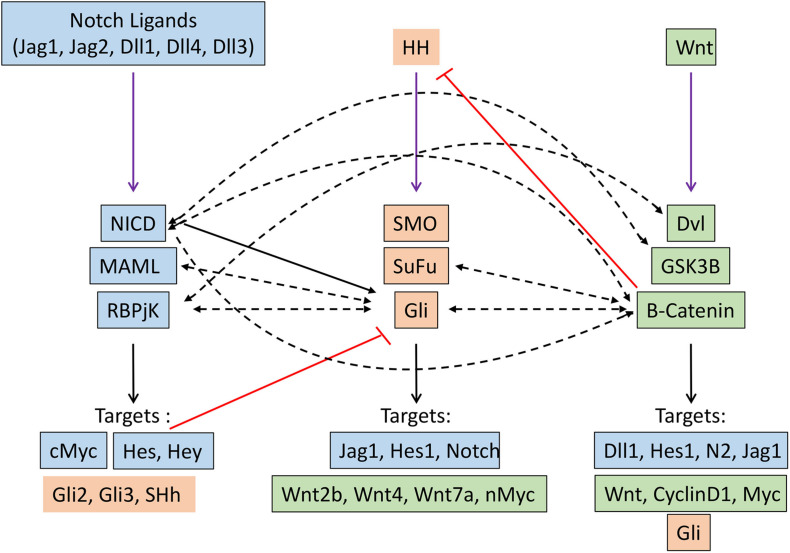
Crosstalk between Notch (blue), Hedgehog (orange) and Wnt (green) signaling pathways in cancer. Black arrow indicates transcriptional regulation. Dashed black arrows indicate direct interactions. Red lines indicate inhibitory regulation.

Several seminal studies over the past decade have demonstrated that Notch, Hh, and Wnt signaling pathways crosstalk with one another, with one pathway acting as an upstream or downstream effector of another. This affects their transcriptional output in the specific cellular context in which they operate (Figure 2).

### Crosstalk Between Notch and Hedgehog Pathway

A genome-wide analysis of the Notch signaling can directly regulate the expression of effector and target molecules of the hedgehog signaling pathway (Li Y. et al., 2012). Hes1, a well-known target of Notch signaling, regulate hedgehog signaling in glioblastoma and thereby therapeutic resistance by directly binding to N-boxes located within the first intron of Gli1 and repressing its expression (Schreck et al., 2010). Notch ligand Jagged1 can regulate hedgehog signaling by downregulating the expression of Gli2, thereby inducing apoptosis and reversing the taxane resistance in ovarian cancer cells both *in vitro* and *in vivo* (Steg et al., 2011). Mastermind-like1 (Maml1), which is a key component of Notch signaling, can act as the regulator of Shh signaling by directly interacting with Gli protein and working as a potent transcriptional coactivator (Quaranta et al., 2017). The indirect regulation of hedgehog signaling by Notch pathway came from the study in Neural stem cells, where the activation of Notch signaling induced the expression of Hes3 and Sonic hedgehog (Shh) through activation of serine/threonine kinase Akt, STST3, and mTOR, this, in turn, promotes the survival of the stem cells (Androutsellis-Theotokis et al., 2006). Moreover, Notch signaling regulates the dynamic localization of the key component of the hedgehog pathway at the primary cilia. This sets the overall cellular threshold for HH responsiveness. In this regard, the activation of Notch signaling results in the accumulation of Smo at the primary cilia, leading to elevated levels of HH response. Interestingly, this Notch-dependent trafficking of Smo to the primary cilia occurs without the presence of the Hh ligand, demonstrating a direct mechanism by which Notch signaling regulates the Hh pathway (Stasiulewicz et al., 2015). Similarly, HH signaling can also directly/indirectly modulate the Notch signaling by controlling the expression of key components of Notch signaling through downstream effectors. The classical Notch target, such as Hes1, can be directly induced by the Hedgehog signaling pathway in a Notch-independent manner through the binding of Gli2 to the promoter of the Hes1 gene (Wall et al., 2009).

### Crosstalk Between Notch and Wnt Pathway

Crosstalk between the Notch and WNT signaling pathways has been elucidated in many developmental processes and also in tumorigenesis. These crosstalks can lead to either feedforward or feedback loop by directly/indirectly regulating the key components of each other. In skin cancer, Notch signaling has been shown to act as a tumor suppressor by inhibiting Wnt signaling. Similarly, in colorectal cancer, Notch1 signaling retained the capabilities of suppressing the expression of Wnt target genes, even when B-Catenin destruction by APC complex was disabled. In addition, Notch can tether B-catenin and thereby modulate its stability; therefore, the Notch1 loss of function leads to the activation of b-catenin (Kwon et al., 2011). Moreover, a negative correlation between Notch1 target gene Notch-regulated ankyrin repeat protein 1 (NRARP) and WNT target genes was found in human colorectal cancer (Kim et al., 2012). In contrast, NARP can act as a positive regulator of Wnt signaling by stabilizing the transcriptional factor LEF1, thereby increasing the LEF1 dependent promoter activity. Recently the role of this crosstalk has been well documented in triggering the early stage of myeloid regeneration and in myeloid malignancies (Kang et al., 2020). On the other hand, Wnt signaling can directly regulate the expression of different components of Notch signaling such as Delta-like1 (Dll1), Hes1, Notch2, Jag1, etc. (Galceran et al., 2004; Ungerback et al., 2011; Li B. et al., 2012). B-Catenin can directly interact with Notch1, resulting in reduced ubiquitination of Notch1, thereby affecting its stability and activity. Similarly, GSK3B also directly interacts with NICD-1 and phosphorylates its serine and threonine residues, thereby affecting its nuclear localization, stability, and transcriptional activities. Moreover, GSK3B can also phosphorylate NICD-2, but this results in reduced transcriptional activity. Dvl, another component of Wnt signaling, physically interacts with RBPJ, resulting in reduce the transcriptional activity of RBPJ, as observed by the promoter activity of Notch responsive reporter construct.

### Crosstalk Between Wnt and Hedgehog Pathway

Gli1/2 induces expression of sFRP-1 (secreted frizzled related protein 1, which negatively regulates Wnt signaling by the subsequent cytoplasmic accumulation of B-catenin (He et al., 2006). Gli1 induces activation of Wnt2b, Wnt4, Wnt7b, which in turn promote the stability of B-catenin, thereby triggering the Wnt signaling (Li et al., 2007). N-myc, which is an important target of the Wnt signaling pathway and is related to medulloblastoma, is regulated by SHH, which promotes expression and post-transcriptional stabilization of N-Myc in mice (Kenney et al., 2003; Thomas et al., 2009). Gli3 physically interacts with the C-terminal domain of B-catenin (the region that includes the transactivation domain), thereby reducing the Wnt-mediated transcriptional activity (Ulloa et al., 2007). Similarly, SuFu is able to bind B-Catenin and export it from nucleus-thus repress B-catenin/TCF mediated transcription. Therefore loss of SuFu leads to increased risk of MB (Taylor et al., 2004). In turn Wnt signaling can modulate hedgehog signaling by directly activating the target gene of hedgehog signaling or modifying the key component of hedgehog singling by directly interacting with them. B-Catenin directly interact with Gli and leads to the proteasomal degradation of GLI, thereby decreases the proliferation of SHH dependant tumors such as MB (Zinke et al., 2015). B-catenin induces the stabilization of Gli mRNAs through upregulating CRD-BP and RNA binding protein (Noubissi et al., 2009). Moreover, it can enhance the luciferase activity of Gli-responsive elements.

## Notch, Hedgehog, and Wnt Signaling in Chemo-Resistance

**FIGURE 3:**
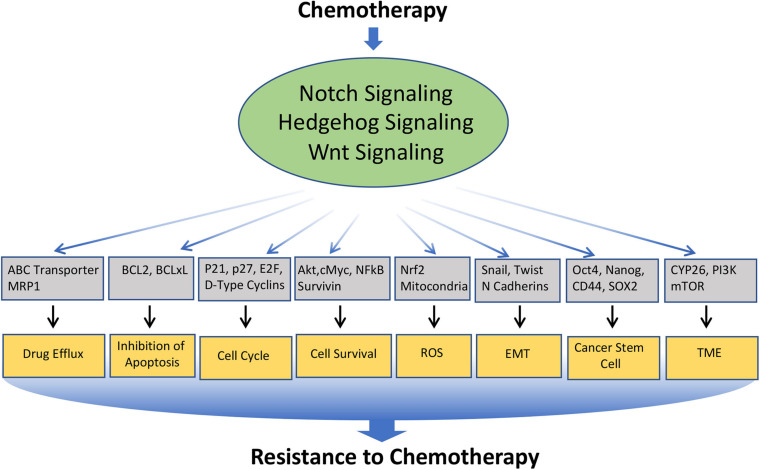
Involvement of Notch, Hedgehog, and Wnt signaling pathways in the resistance of cancer cells to chemotherapeutics drugs: Various anticancer drugs often upregulate Notch, Hedgehog, and Wnt signaling, which in turn regulates the key molecules involved in cellular processes such as Drug efflux, Inhibition to apoptosis, cell survival, cell-cycle, ROS, EMT, CSC, DNA damage response, TME, immune cell functions, etc. that leads to the acquisition of resistance to the chemotherapeutics drugs.

Chemotherapy forms one of the major therapeutic strategies in the treatment for many cancers at different stages of the disease. However, continuous exposure to the chemotherapeutic drug often leads to drug resistance, which is a major problem in cancer treatment. The mechanisms underlying chemoresistance is often complex and multifaceted, which include alteration in drug transport, detoxification of drug, increased or altered drug targets, block in apoptosis, enhanced cell survival, alteration in the cell cycle, induction in epithelial to mesenchymal transition (EMT), enrichment or induction Cancer Stem Cell (CSC) phenotype, modification of the tumor microenvironment, etc. Interestingly, Notch, Hedgehog, and Wnt are involved in modulating nearly all of these mechanisms (Figure 3). In the following section, we will limit our discussion to some of the major mechanisms involved in chemo-resistance, which are modulated by these signaling pathways (Table 1).

**TABLE 1:** Notch, Hedgehog, and Wnt Signaling pathway in resistance of cancer to various chemotherapies.

Cancer type	Chemotherapeutic drug used	Type of resistance	Mechanism	References
**Notch signaling pathway**				
Pancreatic cancer	Gemcitabine	Acquired	Gemcitabine treatment upregulated N2/Jag1 which and induce EMT, leading to develop drug resistance of cells	Wang et al., 2009
	Gemcitabine	Intrinsic	Cancer cells having high Notch3 showed increased resistance to drug, which upon silencing (N3 siRNA) leads to gemcitabine-induced apoptosis.	Yao and Qian, 2010
	Gemcitabine (PDAC)	Intrinsic	Crosstalk between Leptin and Notch signaling regulated the expression of miR343-3p, which inhibited tumor suppressor, KLF6 thereby affecting the chemosensitivity.	Ma et al., 2019
Prostate cancer	Docetaxel	Acquired	Notch signaling is active in stem like cells, which leads to increased expression of ABCB1 (P-glycoprotein), leading to resistance of cells to drug.	Qiu et al., 2018
Breast cancer	Doxorubicin	Intrinsic	DLL1 in tumor stem cells activated Nf-kB survival pathway, which drives chemoresistance in breast cancer.	Kumar et al., 2021
	Doxorubicin	Acquired	Knockdown of Notch via siRNA enhanced sensitivity to Doxorubicin.	Zang et al., 2010
	Trastuzumab	Notch1	Notch-1 could contribute to trastuzumab resistance in breast cancer	Baker et al., 2018
	Paclitaxel	Intrinsic	Hypoxia in tumor microenvironment upregulated HIF2a expression which in turn increased expression of Notch/stem cell marker, leading to enhanced CSC phenotype responsible for resistance to drugs.	Yan et al., 2018
	Cisplatin/doxorubicin	Acquired/Intrinsic	Notch1 bind to promoter of Major Vault protein (MVP) and increases its expression, resulting in activation of AKT mediated EMT, which leads to acquisition of resistance to drugs. Silencing of N1 reversed this effect	Xiao et al., 2019
	Docetaxel	Acquired	Docetaxel activated Notch signaling and suppressed NUMB, which lead to increased survival and EMT acquisition in cancer cells, resulting on enhanced chemoresistance to drugs.	Zhang et al., 2013
Multiple myeloma	Bortezomib, lenalidomide, melphalan	Intrinsic	Jag1 present in stromal cells (niche) activated Notch signaling in MM cells—activates PKC, which phosphorylates MARKS—contributes to survival of MM cells	Colombo et al., 2020
Colorectal cancer	5FU/irinotecan	Intrinsic	ADAM17 inhibitor (ZLDU-8) downregulated Notch signaling leading to decrease EMT, which affected chemoresistance phenotype.	Li et al., 2018
Osteosarcoma	Cisplatin	Intrinsic	Notch inhibition by gamma secretase inhibitor enhances the antitumor effect of cisplatin in resistant osteosarcoma.	Dai et al., 2019
	Methotrexate (MTX)	Intrinsic	Wnt/Notch inhibition with MTX synergistically inhibited growth and increased death of Saos2 cells, thereby sensitizing osteosarcoma cells to chemotherapy.	Ma et al., 2013
Ovarian cancer	Platinum	Intrinsic	Notch3 is upregulated in ovarian cancer stem cells. Combination of cisplatin with GSI effectively eliminates CSCs and bulk tumors, thus sensitizes tumors to platinum therapy	McAuliffe et al., 2012
	Platinum/Taxane	Intrinsic	Upregulation of Notch 3 was observed in drug resistant cell. Silencing of Notch3 using siRNA induced apoptosis in resistant cells	Rahman et al., 2012
Epithelial ovarian cancer cells	Cisplatin	Acquired	Nuclear orphan receptor NR2F6 was upregulated in resistant EOC, which leads to sustain expression of Notch3 signaling in Cancer Stem Cells, leading to acquisition of resistant phenotype.	Li Q. et al., 2019
GBM	Temozolomide	Acquired	Loss of PLK2 leads to activation of Notch signaling in GBM, which induces the acquired resistance to Temozolomide	Alafate et al., 2020
	Etoposide	Intrinsic	Notch2 sig enhances FGFR1 activity to target AKT-GSK3 signaling to block apoptosis	Tome et al., 2019
ESCC (esophagous squamous cell carcinoma)	Chemoresistance	Intrinsic	Activation of Notch/Wnt signaling by PRMT1 in tumor initiating cells was responsible for chemoresistance phenotype.	Zhao et al., 2019
hepatocellular carcinoma (HCC)	vincristine and 5-fluorouracil	Intrinsic	In CD133+ HCC cells, increased activation of Notch signaling is observed, and its inhibition enhanced BBC3-mediated apoptosis leading to increased sensitization of cells to vincristine and 5-fluorouracil	Hemati et al., 2020
Acute myeloid leukemia (AML)	Adriamycin	Acquired	Chemoresistant AML cells showed increased expression of PRKD2, which regulated Notch signaling pathway	Liu et al., 2019
T cell acute lymphoblastic leukemia.	Glucocorticoid	Acquired	Activated Notch signaling regulate AKT, which resulted in survival and resistance to glucocorticoids.	Piovan et al., 2013
**Hedgehog signaling pathway**				
Cancer	Chemotherapeutic drug used	Type of resistance	Mechanism	
Pancreatic cancer	5FU and gemcitabine	Intrinsic	TET1 downregulated the CHL1-associated Hedgehog signaling pathway, thereby reverses chemoresistance in PDAC.	Li H. et al., 2020
Pancreatic cancer	Gemcitabine	Intrinsic	HH signaling is responsible for chemoresistance in pancreatic cancer and its inhibition in combination with CXCR4 inhibition improved chemotherapeutic efficacy in pancreatic cancer.	Khan et al., 2020
Breast cancer	Docetaxel	Intrinsic	Neoplastic cells secrete Hedgehog ligands to modify CAF, which in turn secrete cytokines for CSC and collagen fibrillar to modulate microenvironment, thus providing chemoresistance to drugs.	Cazet et al., 2018
Multiple myeloma	Bortezomib	Intrinsic	Gli2 was restricted in high acetylation and low ubiquitination states in bortezomib resistant mylenoma cells, thererby upregulating Hedgehog signaling in stem cells, which are resistant to drugs.	Xie et al., 2020
	Bortezomib	Intrinsic	HH secretion by MM cells upregulated stromal CYP26 and further reinforced a protective microenvironment against drugs	Alonso et al., 2016
Colorectal cancer	Chemotherapy	Intrinsic	HIF-1α and cancer-associated fibroblasts (CAFs)-secreted TGF-β2 activate the expression GLI2 in CSCs, resulting in increased stemness/dedifferentiation and intrinsic resistance to chemotherapy	Tang et al., 2018
	5-FU/tirinotecan	Intrinsic	HH inhibitor (AY9944/GANT61) with 5FU/Trinotecan reduces stem cells marker and colony formation, thereby reduces resistance to chemotherapy.	Usui et al., 2018
GBM	Temozolamide	Intrinsic	Shh/Gli1 regulated BMI, which in turn regulated MRP1, thereby chemoresistance. Thus inhibition of Hedgehog by (GANT61) together with Temozolomide showed synergistic effect.	Shahi et al., 2016
	Temozolamide	Intrinsic	Gli inhibition modulates nuclear p53 levels and decreases MGMT expression in combination with TMZ, leading to increases apoptosis, and decreases stem like cells, thus affecting chemoresistance to drugs	Melamed et al., 2018
	Temozolamide	Intrinsic	Hedgehog signaling directly regulate MGMT expression and chemoresistance to TMZ. Thus inhibition of Hedgehog activity restored the chemosensitivity to TMZ	Wang et al., 2017
Acute myeloid leukemia	Adriamycin	Acquired	Drug resistant cells have increased HH signaling. Inhibiting of Hedgehog signaling by NVP-LDE225 lowers MRP1 expression, leading to increased intracellular accumulation of Adriamycin, thereby reversing the chemotherapeutic resistance.	Huang K. et al., 2019
Hepatoma cells	Itraconazole	Intrinsic	CSC has aberrant activation of Hedgehog signaling and regulate the drug sensitivity of hepatoma through the ABCC1 transporter.	Ding et al., 2017
Gastric cancer	Cisplatin	Acquired	Treatment with cisplatin upregulate Hedgehog signaling in CSC, which directly upregulate ABCG2 expression, which leads to acquisition of chemoresistance to drug.	Yu et al., 2017
NSCLC	Platinum	Intrinsic	Gli2 expression in NSCLC, provide resistance to platinum based chemotherapy.	Giroux Leprieur et al., 2016
Oral squamous cell carcinoma (OSCC)	5-fluorouracil (5-FU) and Cisplatin (CDDP)	Acquired	Activation of Hedgehog signaling pathway in oral squamous cell carcinoma, regulate ABC transporters, which is associated with Multidrug resistance in OSCC	Lu et al., 2020
Colorectal cancer (CRC)	5-fluorouracil and Oxaliplatin	Acquired	Gli1 regulates the ABC transporters expression, thereby promoting the chemoresistance features.	Po et al., 2020
Epithelial ovarian cancer	Paclitaxel, Doxorubicin, Cisplatin	Intrinsic	Sub population expressing high Gli1 showed chemoresistance, which was mediated by regulating ABC transporter.	Chen et al., 2014
**Wnt signaling pathway**				
Cancer	Drug	Type of resistance	Mechanism of action	
Pancreatic cancer	Gemcitabine	Intrinsic	Overexpression of long noncoding RNA PVT1 and Pygo2 is associated with high Wnt/B- activation	Zhou et al., 2020
High-grade serous ovarian cancer (HGSOC)	Platinum	Intrinsic	miR-181a-SFRP4 axis mediates Wnt activation and promotes stemness and cell proliferation	Belur Nagaraj et al., 2021
Cholangiocarcinoma (CCA	Gemcitabine	Acquired	lncRNA LINC00665 expression is associated with BCL9L or miR-424-5p—Wnt/b-catenin signaling via regulating stemness and EMT in drug resistant cells	Lu et al., 2021
Oral squamous cell carcinoma (OSCC)	Cisplatin	Acquired	ENO/AKT/GSK3b axis drives CMTM6 mediated resistance with enhanced activation of Wnt signaling in cisplatin resistant cells by modulating apoptosis mediated cell death	Mohapatra et al., 2021
Colorectal Cancer	5-Fluorouracil (5-FU) /Oxiplatin	Acquired	Increased MUC5AC expression mediated by CD44/β-catenin/p53/p21 signaling in drug resistant cells	Pothuraju et al., 2020
Leukemia stem cells	Doxorubicin	Acquired	Low dose of DOX inhibits Akt-β-catenin interaction by inhibiting the expression of immune checkpoints like PD-1,TIM-3, and CD24	Perry et al., 2020
Non-small cell lung carcinoma (NSCLC)	Erlotinib	Acquired	Wnt pathway contributes to Erlotinib resistance by lowering the LHX6 expression with reduced cell migration	Wang Q. et al., 2020
Glioblastoma	Temozolomide	Acquired	miR-181c/RPN2/wnt/β-catenin signaling axis	Sun et al., 2020
Triple negative breast cancer	*Cis*-Platin	Acquired	FZD8-mediated Wnt-signaling mediating CSCs growth and resistance to chemotherapy and its inhibition enhances the chemotherapeutic response in TNBC and induces cell death and inhibits cell growth	Venkatesh et al., 2020
Acute lymphoblastic leukemia (ALL)	Dasatinib	Acquired	Transcriptional coactivator CBP increased dasatinib sensitivity in Wnt and Pre BCR dependent pathway	Duque-Afonso et al., 2018
Pancreatic adeno carcinoma	Gemcitabine	Acquired	Fam83D high expression is associated with gemcibine resistance in pancreatic cancer cells (PDAC) and proliferation, mitochondrial respiration capacity, aerobic glycolysis, C-Myc	Hua et al., 2021
CLL	Lenalidomide	Acquired	Restrained activation of Wnt signaling and sensibility to lenalidomide is associated with SHISA3 reduced apoptosis	Maffei et al., 2018
Soft tissue sarcoma	Doxorubicin	Acquired	PRI 724+Dox inhabited the Wnt signaling via CDC25A and CCND1 reduced expression. This leads enhanced cell cycle arrest and reduced cell proliferation	Martinez-Font et al., 2020
Lung adenocarcinoma cell	*Cis*-platin	Acquired	β-Catenin signaling pathway regulates cisplatin resistance in lung adenocarcinoma cells by upregulating Bcl-xl	Zhang J. et al., 2016
Neuroblastoma	Doxorubicin	Acquired	FZD1 mediates chemoresistance in neuroblastoma through activation of the Wnt/beta-catenin pathway by increasing cell proliferation and survival.	Flahaut et al., 2009

### Drug Efflux

Drug efflux forms the primary mechanism of chemo-resistance in cancer. Emerging evidences suggest that Notch, Hedgehog, and Wnt signaling can directly modulate the drug efflux by regulating the expression of the transporter involved in drug efflux.

In prostate and breast cancer, the chemotherapeutic drug such as docetaxel or doxorubicin induces the activation of Notch signaling, which in turn increases the expression of ABCB1 and multidrug-resistant associated Protein1 (MRP1), thus increasing the drug efflux and contributing to chemo-resistance. This chemo-resistance effect is reversed by the Notch inhibitors (Zhang et al., 2013; Qiu et al., 2018).

Likewise, Hedgehog signaling directly regulates the expression of ABCB1 and ABCG2 in ovarian cancer. The inhibition of Gli1 expression decreases ABCB1 and ABCG2 gene expression levels, thus enhancing the response of ovarian cancer cells to certain chemotherapeutic drugs (Chen et al., 2014). Moreover, Hedgehog signaling transcription factor Gli1/Gli2 appears to be the primary regulator of drug response in hepatoma, Oral squamous cell carcinoma (OSCC) and Colorectal cancer (CRC) through the ABC transporter and in Acute Myleiod Leukemia and GBM through MRP1 (Shahi et al., 2016; Ding et al., 2017; Huang K. et al., 2019; Lu et al., 2020; Po et al., 2020).

Similarly, the Wnt/β-catenin pathway is known to regulate the transporters involved in drug efflux. Several TCF4/LEF binding motifs are present in the promoter region of the ABCB1 gene in humans, suggesting it as a target gene of the β-catenin/TCF4 transcriptional regulators, thus the activation of β-catenin augments ABCB1 expression. LGR5, the target molecules for the Wnt pathway in colorectal cancer cells, was found to confer resistance to chemotherapy. ABCB1 higher expression was found to associated LGR5 expression in chemoresistance to CRCs. Cancer stem cell property in CRCs is regulated by LGR5 that leads to chemoresistance to oxaliplatin and 5-fluorouracil (Liu et al., 2013). Pygopus (PYGO2) is involved in the signal transduction of the Wnt pathway and plays a critical role in the development of tumors. It is also linked with Multi-Drug Resistance in various cancers like breast, ovarian, lung, glioma, and esophageal squamous cell carcinoma. This is also one of the most up-regulated genes in chemo-resistant breast cancer. Increased expression of the PYGO2 gene upregulates the ABCB-1 gene in the resistant cells through Wnt/β-catenin pathway (Zhang Z.M. et al., 2016). CD44 is a surface protein that plays an important role in intercellular communication within the tumor microenvironment. CD44 upregulates the expression of ABC transporters, thereby inducing a form of drug resistance known as cell adhesion-mediated drug resistance (CAM-DR) (Martin-Orozco et al., 2019). CD44 has been identified to be responsible for lenalidomide resistance mediated by the Wnt cascade. Overexpression of CD44 has been observed in lenalidomide-resistant human multiple myeloma cell lines (HMCLs), with increased adhesion to bone marrow (BM) stromal cells, while inhibition of CD44 reduced the adhesion of multiple myeloma cells and reversed the resistance to lenalidomide (Spaan et al., 2018).

### Inhibition of Apoptosis

Down-regulation of apoptosis induced by chemotherapeutic drugs is another mechanism that contributes to chemo-resistance in cancer. Events that lead to a block in apoptosis enhances chemo-resistance. Notch, Hedgehog, and Wnt signaling have been well known to modulate apoptosis in cancer.

Notch signaling regulates apoptosis through its interaction with the key players of apoptosis pathways (Lundell et al., 2003; Zweidler-McKay et al., 2005). Therefore, blocking Notch signaling has often been found to result in the induction of apoptosis in cancer. In platinum/Taxane resistant ovarian cancer, Notch3 has been found to be upregulated, and downregulation of Notch3 by siRNA or GSI induces apoptosis in resistant cells (Rahman et al., 2012). In pancreatic cancer, the subpopulation having high Notch3 showed increased resistance to Gemcitabine, which upon silencing (N3 siRNA) leads to induced apoptosis (Yao and Qian, 2010). Similarly, in glioblastoma, osteosarcoma and hepatocellular carcinoma (HCC) inhibition of Notch signaling induces apoptosis in the cells that are resistant to chemotherapeutic drugs like Temozolamide, Etoposide, Cisplatin, and vincristine and 5-fluorouracil (Dai et al., 2019; Tome et al., 2019; Alafate et al., 2020; Hemati et al., 2020).

Hedgehog signaling pathways has also been implicated in the regulation of apoptosis in cancer (Athar et al., 2004). Emerging evidence suggests that Hedgehog regulated apoptosis in cancer may play an essential role in the acquisition of chemo-resistance. Consequently, the treatment of glioblastoma with temozolomide together with an inhibitor of Gli induces apoptosis, thereby reducing the chemo-resistance (Melamed et al., 2018).

Several inhibitors that target apoptosis regulation by modulating Wnt signaling have been investigated. For example, Belinostat is a histone deacetylase inhibitor that induces apoptosis by decreasing the Wnt/β-catenin, CCND2, and Myc in MCF-7 cells (Lu et al., 2019). Lanatoside C inhibits Wnt/β-catenin signaling by down-regulating c-Myc in gastric cancer cells, while overexpression of c-Myc reverses the anti-tumor effect of lanatoside C, suggesting that c-Myc is a key drug target of lanatoside C (Hu et al., 2018). Wnt inhibitor FH535 has been found to markedly suppress the expression of β-catenin target genes (LEF1, CCND1, and cMYC) and potentiate imatinib-induced apoptosis (Suknuntha et al., 2017). FH535 also shows antiproliferative effects in leukemia cell lines like THP-1, Jurkat, HL60, and K562 (Suknuntha et al., 2017). Coenzyme Q0 (CoQ0; 2,3-dimethoxy-5-methyl-1,4-benzoquinone), a novel quinone derivative, showed the *in vitro* and *in vivo* anti-tumor, apoptosis, and anti-metastasis activities of CoQ0 (0–20 μM) through inhibition of Wnt/β-catenin signaling pathway.

### Cell Cycle

In the recent past, it has become increasingly apparent that the cell cycle plays a critical role in chemosensitivity for the given chemotherapy, and the role of developmental pathways such as Notch, Hedgehog, and Wnt signaling in this phenomenon has been well established (Arora and Spencer, 2017).

Notch signaling regulates the cell cycle by inducing the expression of some of the critical cell-cycle related genes such as p21, p27, E2F etc. Therefore cell-cycle perturbation due to dysregulated Notch signaling in cancer forms one of the factors that impart chemoresistance to the drugs. For example, in T-cell transformation and chemoresistance, Notch1 signaling is associated with the promotion of G1-S through upregulation of CDK4 and CDK6 and downregulation of p27/KIP1 and p18/INK4C cell cycle inhibitors (Joshi et al., 2009). Therefore inhibition of Notch signaling by pharmacological or genetic approach leads to cell cycle arrest and apoptosis in cancer cells (Li et al., 2014). Similarly, in Prostate cancer stem-like cells (PCSCs), the combination of GSI with DOX promoted DOX-induced cell growth inhibition, apoptosis, cell cycle arrest, and sphere formation in PCSCs when compared to DOX only. This suggest that Notch inhibition may have clinical benefits in targeting PCSCs to enhance the anti-tumor effect of DOX in PC-3 PCSCs (Wang L. et al., 2020).

Similarly, the Hedgehog signaling pathway modulates the cell cycle progression via directly regulating the core cell cycle components such as D-type cyclins, Cip1, p57KIP2 (Neumann, 2005; Adolphe et al., 2006). Moreover, the role of cell cycle regulation by the hedgehog signaling pathway in the acquisition of chemoresistance is also well documented. Therefore, the inhibitors of the hedgehog pathway together with the chemotherapeutic drug are being explored to circumvent the chemotherapy acquired during the treatment. For example, in GBM, the combination of Hedgehog inhibitor GANT-61 with Temozolomide showed a synergistic effect, and all TMZ- resistant cell lines displayed a significant decrease in cell viability due to arrest in G2/M and increase apoptosis (Honorato et al., 2020). The combination of bortezomib with hedgehog antagonist, LDE225, increased paclitaxel sensitivity through apoptosis and G2/M arrest in ovarian cancer. Thus the inhibition of protease inhibition and Hedgehog signaling can reverse taxane-mediated chemoresistance (Steg et al., 2014).

Wnt/β−catenin signaling regulates cell proliferation by modulating the cell cycle via β -catenin, which is a well-known regulator of cell cycle progression (Nelson and Nusse, 2004). Moreover, it has been well established that a tight control of β catenin levels are required for cell cycle progression (Olmeda et al., 2003). In cancer cells, the Wnt-mediated cell cycle regulation has been implicated in imparting resistance to chemotherapy. Thus various inhibitors of Wnt signaling are being explored to attenuates the chemoresistance developed by the cancer cells. In human neuroendocrine tumor (NET) cell lines Porcupine (PORCN) inhibitor WNT974 and the β-catenin inhibitor PRI-724 resulted in the cell cycle arrest at the G1 and G2/M phases, which affected the tumor growth and viability (Jin et al., 2020).

### Cell Survival

Cell survival forms an important factor that attenuates the effectiveness of conventional chemotherapy. The role of Notch, Hedgehog, and Wnt signaling in regulating cell survival in the face of chemotherapy treatment has gained much interest due to its therapeutic importance.

Report from multiple laboratories has shown the involvement of Notch signaling in the regulation of cell survival in normal development and also in cancer. Moreover, recent reports suggest that Notch signaling form the key mediator of increased cancer cell survival in the context of chemotherapy. For example, activated Notch protected T-ALL cells from glucocorticoid-mediated apoptosis, which was mediated from Notch dependent upregulation of Akt, leading to nuclear export of glucocorticoid receptor (Piovan et al., 2013). Thus the combined use of GSIs with glucocorticoids in patients with relapsed/refractory T-ALL showed a promising result in the clinical trials, where γ-Secretase inhibitors reverse glucocorticoid resistance in T cell acute lymphoblastic leukemia. In addition, the critical role of Notch signaling in tumor cell survival and apoptosis resistance was shown in B- CLL (Rosati et al., 2009). Similarly, the breast cancer cells which expressed a high level of Jagged1 shown resistance to lapatinib and increased survival with enhanced tumor-initiating potential (Shah et al., 2018). Another study reported that Notch1 mediated repression of PTEN in HER2+ breast cancer cells and was responsible for trastuzumab resistance through increased survival of breast cancer cells (Baker et al., 2018). Similarly, the Dll1, Jag1 mediated survival is responsible for chemoresistance in breast cancer and multiple myeloma.

Hh signaling also has been shown to promotes cancer cell survival, which forms a selective growth advantage to tumor cells and has been implicated in multidrug resistance. In rhabdomyosarcoma (RMS) or Ewing sarcoma (EWS), HH pathway activity and GLI1 expression contribute to cell survival and proliferation. Chemotherapeutic drugs such as Vincristine (VCR), was shown to significantly upregulate Gli1 expression in these cells. Thus treatment with small molecule inhibitor GANT61 or siRNA against GLI1 together with vincristine significantly decreased cell viability (Yoon et al., 2020). Similarly, Pemetrexed resistant NSCLC cells showed significantly increased expression of HH signaling genes (GLI1, GLI2, GLI3, PTCH1, SHH). Supporting these results, pemetrexed resistant cells treated with the HH inhibitor Gant61 showed reduced proliferation and survival compared to naïve cells. Thus, blocking the HH pathway may be a potential option to overcome resistance to various chemotherapy (Liu et al., 2020).

Similarly, Wnt signaling is involved in regulating the drug resistance of various cancer via cell survival and proliferation. Thus inhibiting Wnt signaling can sensitizes cancer cells to chemotherapy. For example, in myeloid leukemia cells, the Wnt inhibitor (FH535) sensitizes it to chemotherapeutic drug imatinib and potentiated its chemotherapeutic effect (Suknuntha et al., 2017). A similar effect of Wnt inhibitor was observed in ovarian cancer cells, where the inhibition of the Wnt pathway reversed the drug resistance by inducing apoptosis and reducing the cell survival. TNBC cells and tissues resistant to chemotherapy shows sensitivity toward Wnt mediated ST8SIA1 expression. The reduced proliferation and cell survival were observed in chemo-resistant cells with ST8SIA1 inhibition. ST8SIA1 inhibition is associated with the suppression of FAK/Akt/mTOR and Wnt/β-catenin signaling pathways (Wan et al., 2020).

### Reactive Oxygen Species (ROS)

ROS plays a crucial role in cancer progression and resistance to radio-and chemotherapy (Cui et al., 2018). Their role in modulating Notch, Hedgehog, and Wnt signaling and vice-versa has been investigated in different cancer that leads to the development of resistance to therapy.

Notch and ROS can modulate each other by directly regulating their core components. Nrf2 is a well-established regulator of anti-oxidants and its overexpression cause resistance to both radio-and chemo-therapy in cancer (Tian et al., 2017). Notch can directly bind to the promoter region of Nrf2 and regulate its transcription, thereby affecting the ROS level in the cells (Wakabayashi et al., 2014). Notch signaling has also been suggested to alter the proteome of mitochondria, which results in an alteration of its function. As Mitochondria are considered as the major source of ROS in cancer (Hayes et al., 2020), the alteration of their function by Notch can have a prominent role in the acquisition of chemoresistance. Moreover, the Notch pathway was shown to be critical for controlling the ROS level in CSCs, thereby affecting the chemoresistance (Qiang et al., 2012).

Similarly, the hedgehog signaling pathway can crosstalk with ROS-induced signaling to regulate chemoresistance in various cancer. For example, in hepatocellular carcinoma HCC cells, ROS-induced NRF2 activation upregulated the expression of sonic hedgehog homolog (SHH), ultimately activating sonic hedgehog pathway. This mediated the tumor initiating function, ultimately leading to sorafenib resistance in HCC. Moreover natural compound such as, resveratrol and curcumin was shown to inhibit hypoxia-mediated activation of the Hh signaling pathway in pancreatic cancer, ultimately affecting the EMT phenotype, which has been known to play an important role in inducing chemoresistance in cancer (Cao et al., 2016; Li et al., 2016). In contrast to the activation of hedgehog in tumor cells, activation of the canonical Hh signaling pathway in stromal cells by IHH was shown to suppress tumor growth and metastases, in part, by limiting ROS activity (Kasiri et al., 2020).

The interplay between Wnt signaling and ROS has investigated in different cancer that leads to the development of resistance to therapy. Activation of Wnt canonical signaling aids the ubiquitination and proteasomal degradation of Nrf2 protein via axin1/GSK-3b complex (Tian et al., 2017). ROS/Wnt/β-catenin signaling has been shown to regulate the stemness in hepatocellular carcinoma (HCC) by targeting glutaminase 1 (GLS1) (Li B. et al., 2019). Thus, targeting GLS1 attenuates stemness properties in HCC by increasing ROS and suppressing Wnt/β-catenin pathway and hence, GLS1 served as a therapeutic target for the elimination of CSCs. Recently, mitochondrial ROS NF-kB/b catenin axis was found to play significant role in regulating gall bladder cancer. Moreover, ROS mediated alteration in Wnt signaling impacts the vascular development that constitutes changes in stem cell differentiation, angiogenesis, VEGF signaling, endothelial as well as cardiac progenitor cell recruitment, and vascular cell migration in cancer and cardiovascular diseases (Caliceti et al., 2014). Thus, targeting Wnt signaling with inhibitors that curb ROS may synergistically overcome the chemoresistance in various cancers.

### Induction in Epithelial to Mesenchymal Transition (EMT)

Epithelial to mesenchymal transition (EMT) is a process where the epithelial cells lose their epithelial characteristics and acquire mesenchymal characteristics. This dramatic cell transposition process not only plays critical roles in governing embryonic development and maintaining adult tissue hemostasis (e.g., via regulating wound healing and stem cell behavior) but also contributes to pathological conditions, such as fibrosis, cancer progression, and drug resistance.

Notch signaling can directly regulate the expression of genes involved in EMT (Nieszporek et al., 2019). It is increasingly recognized that the Notch signaling is involved in the acquisition of EMT in drug-resistant cancer cells. In gemcitabine-resistant pancreatic cancer cells, Notch-2 and Jagged-1 are highly up-regulated, and downregulation of Notch signaling by siRNA leads to a partial reversal of the EMT phenotype, resulting in the mesenchymal-to-epithelial transition (MET), which is associated with decreased expression of vimentin, ZEB1, Slug, Snail, and NF-κB (Wang et al., 2009). A similar effect has also been observed in breast cancer cells resistant to cisplatin/doxorubicin/Docetaxel, where the inhibition of Notch signaling leads to a decrease in EMT, thus sensitizing them to the chemotherapeutic drugs (Zhang et al., 2013; Xiao et al., 2019). In colorectal cancer, ADAM17 inhibitor (ZLDU-8) downregulated Notch signaling leading to decrease EMT, which affected chemoresistance phenotype (Li et al., 2018).

The role of the Wnt/β-catenin pathway has also been implicated in the EMT process. Runt-related transcription factor 1 (RUNX1) plays the roles of an oncogene and an anti-oncogene in epithelial tumors, and abnormally high expression of RUNX1 is associated with metastasis and EMT. It directly interacts with catenin and targeting the promoter and enhancer regions of KIT (Li Q. et al., 2019). Further, the expression of TRIM29 (Tripartite Motif 29) has been reported to be influenced by Wnt/β-catenin. TRIM 29 can activate this pathway in colorectal cancer (CRC) cells by promoting metastasis and invasion by regulating EMT through increased expression of CD44. Rhomboid domain 1 (RHBDD1) containing has also been implicated in metastasis and invasion of CRC mediated by the Wnt/β-catenin pathway, as the expression of RHBDD1 strongly correlates with ZEB-1 in lymphatic and distal metastasis (Pothuraju et al., 2020).

### Enrichment/Induction of Cancer Stem Cell Like Phenotype

Cancer Stem Cell (CSC) is a small subset of tumor cells that has the potential to self-renew and give rise to a heterogeneous population in the tumor. Many studies have demonstrated that CSC is more resistant to chemotherapy because of the higher expression of the anti-apoptotic protein, multidrug resistance gene, etc. These studies have been extensively reviewed elsewhere (Shibue and Weinberg, 2017). The role of Notch, Hedgehog, and Wnt signaling in CSC function in various cancers has been well established (Oren and Smith, 2017; Yang et al., 2020). It is now well appreciated that inhibition of these signaling pathways can reduce the CSCs/stem-like properties, thereby enhancing the chemosensitivity toward various drugs.

The Numb-/low population of castration-resistant prostate cancer has been shown to up-regulate Notch and Hedgehog signaling. This is associated with increased expression of genes linked to stem cells and greater resistance to androgen deprivation therapy (ADT). Inhibition of Notch and Hedgehog signaling pathways significantly increases apoptosis in Numb-/low cells in response to ADT (Guo et al., 2017). Consequently, overexpression of Notch3 leads to the expansion of CSCs, resulting in increased resistance against platinum in ovarian cancer. At the same time, Notch inhibition by pharmacological or genetic approaches depletes CSCs, thereby increasing tumor sensitivity to platinum (McAuliffe et al., 2012). Similarly in Epithelial Ovarian cancer and esophagous squamos cell carcinoma (ESCC) cells, sustain expression of Notch signaling in CSCs/tumor initiating cells, leads to acquisition of drug resistant phenotype (Li H. et al., 2019; Zhao et al., 2019).

In glioblastoma, CD133^+^ CSCs express higher levels of miR-9 and activate the SHH/PTCH1/MDR1 axis, which imparts resistance against TMZ (Munoz et al., 2015). Hedgehog pathway appears to transcriptionally regulate the expression of twist1 and snail in the acquired chemo-resistant cancer cells and chemo-sensitive squamous carcinoma (KB) cancer cells, thereby maintaining the tumor-initiating cell-like properties and consequently the chemo-resistant phenotype, which is independent of ABC transporters (Kong et al., 2015).

Likewise, the Wnt signaling also plays a prominent role in maintaining CSCs which is linked to drug resistance. In cancer such as pancreatic cancer, lung adenocarcinomas, colorectal cancer, and liver cancer, the small subpopulation of cancer cells expressing high Wnt/β-catenin signaling showed CSCs phenotype and were resistant to drug. The inhibition of Wnt signaling in these cells suppressed the stem cells phenotype, thereby affecting the chemoresistance (Taelman et al., 2010; Ilmer et al., 2015; Belur Nagaraj et al., 2021).

### Modification of the Tumor Microenvironment

Increasing evidence suggests that the tumor microenvironment plays a critical role in cancer drug resistance. Apart from playing an essential role in cancer cells, Notch, Hedgehog, and Wnt signaling pathways have prominent roles in modifying tumor microenvironment, which has been implicated in providing chemo-resistance.

The Notch signaling can induce the differentiation of the neighboring cancer cells, which can adopt different cell fate, thus creating a heterogeneous population. The heterogeneity in the tumor is one of the major contributors to the acquisition of chemoresistance (Lim et al., 2017). Furthermore, Notch ligands present in the components of the tumor microenvironment, such as endothelial cells, fibroblasts, immune cells, etc., can induce Notch signaling in cancer cells leading them to acquire stem cell phenotype and becoming resistance to chemotherapy (Boelens et al., 2014; Cao et al., 2014).

In breast cancer, the hedgehog ligand secreted by neoplastic cells modifies CAF, which in-turn provides a supportive microenvironment for the acquisition of a chemo-resistant CSC phenotype via FGF5 expression and production of fibrillar collagen. Consequently, treatment with smoothened inhibitors (SMOi) down-regulates the expression of CSC markers and sensitizes tumors to chemotherapy drugs such as docetaxel (Cazet et al., 2018). Hedgehog secretion by multiple myeloma cells has been found to upregulate stromal CYP26 and further reinforce a protective microenvironment. These results suggest that crosstalk between Hedgehog and retinoid signaling can modulate the tumor microenvironment and cause resistance against anticancer therapeutics (Alonso et al., 2016). Along with other signaling pathways like Notch, TGF-b and receptor tyrosine kinase, the Wnt signaling pathway is also been known to play an important role in inducing EMT in tumor cells (Jing et al., 2011).

Likewise, the Wnt ligand or the growth factor secreted by stromal cells of TME leads to the stimulation of Wnt signaling in tumor cells, that leads to the EMT (Patel et al., 2019). For example, in colorectal cancer cells (CRCs), stimulation of hepatocyte growth factor (HGF) upregulates the β-catenin expression through the PI3-K pathway (Fodde and Brabletz, 2007). This leads to the initiation of the EMT process, which mediates the chemoresistance.

## Notch, Hedgehog, and Wnt Signaling in Radio-Resistance

**FIGURE 4:**
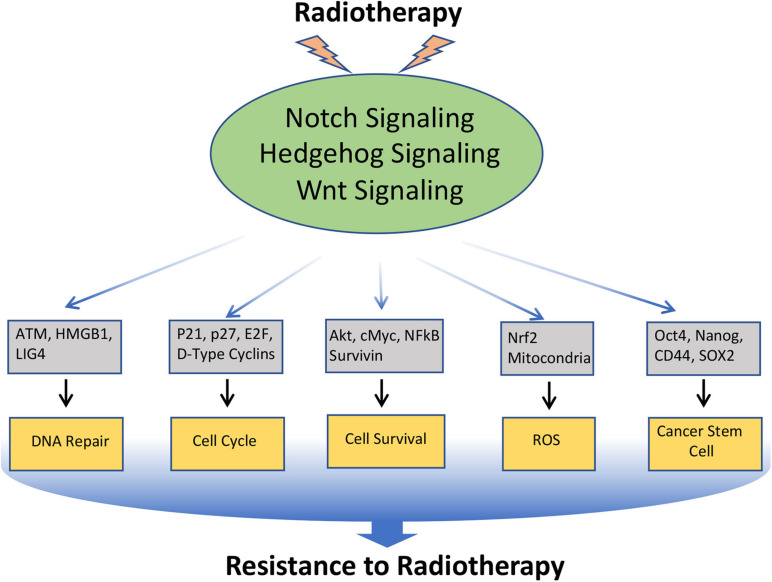
Involvement of Notch, Hedgehog, and Wnt signaling pathways in the resistance of cancer cells to Radiotherapy: Radiotherapy often induces Notch, Hedgehog, and Wnt signaling in cancer, which in turn regulates the key molecules involved in DNA repair, cell-cycle, cell survival, ROS generation, EMT, and CSC, that leads to the acquisition of radioresistance in cancer.

Radiotherapy is used in the treatment of approximately 50% of all malignancies. Resistance to radiation therapy is polymodal and is associated with several biological alterations both within the tumor and in the surrounding microenvironment. Moreover, like chemotherapy, radiotherapy also induces EMT and CSC-like phenotype in cancer cells, which contributes to the radio-resistance. As discussed earlier, Notch, Hedgehog, and Wnt have a prominent role in EMT and CSC, and inhibition of these pathways enhances radio-sensitivity through down-regulation of EMT and CSC (Yahyanejad et al., 2016; Figure 4).

### Notch Signaling in Radioresistance

It has now been well established that Notch signaling gets activated upon irradiation and plays an important role in the radio-resistance of both stem and non-stem cancer cell populations (Yahyanejad et al., 2016). Moreover, in many radio-resistant tumor and cancer cell lines, the Notch signaling is found to be upregulated. Notch signaling can regulate the DDR by directly interacting with ATM and inactivating its kinase activity (Vermezovic et al., 2015; Adamowicz et al., 2016). In line with this, an inverse correlation between Notch and pATM has been found in breast cancer, and Notch inhibition results in increased radiation sensitivity in an ATM-dependent manner. Notch signaling can also affect the radio-resistance by modulating the function/activity of other signaling molecules involved in cell survival, metabolism, cell cycle, etc. In glioma stem cells (GSCs), inhibition of Notch with γ-Secretase Inhibitors (GSIs) does not alter the DNA Damage Response of GSCs but rather suppresses the Akt activity and Mcl1 level after radiation, making GSCs more sensitive to radiation at clinically relevant doses (Wang et al., 2010). Further, ALDH positive breast cancer cells are more radio-resistant as compared to the ALDH negative counterpart. Mechanistic studies have shown that radio-resistance linked to ALDH activity stimulation is mediated through the activation of the Notch1 and AKT pathways and involves Nanog signaling. Nrf2 plays a major role in regulating the cellular antioxidant system and is activated by radiation in a dose-dependent manner. A decrease in Nrf2 expression significantly dampens Notch1 expression following ionizing radiation and potentiates IR-induced apoptosis. Nrf2-mediated Notch signaling has been an important determinant in the radio-resistance of lung cancer cells, while TRIB3 activates the Notch signaling in radio-resistant triple-negative breast cancer.

### Hedgehog Signaling in Radioresistance

The role of Hedgehog signaling in the radioresistance of tumors has been well established. GLI proteins are the functional transcription activators of the Hh pathway, and the Inhibition of its activity can interfere with almost all DNA repair types in human cancer, indicating that Hh/GLI functions may play an important role in radiation-induced DNA damage (Meng et al., 2015). Indeed, Hedgehog signaling is found to be upregulated/activated following irradiation, and this has been implicated in providing radio-resistance. Consequently, the inhibition of hedgehog signaling augments the efficacy of radiation in tumors that are dependent on hedgehog signaling (Teichman et al., 2018). In HNSCC, Gli1 is often upregulated at the tumor-stroma intersection, which gets further augmented following irradiation, where it contributes to stromal-mediated radio-resistance of the tumor. Treatment with HH inhibitors has been found to enhance tumor sensitivity to radiotherapy (Gan et al., 2014). Further, intrinsic or acquired radio-resistance of tumors is often associated with up-regulated Hedgehog signaling, and the downregulation of this by either pharmacological inhibition or genetic manipulation renders them sensitive to radiation.

### Wnt Signaling in Radioresistance

WNT signaling has also been implicated in the radioresistance of cancer cells. An increase in the WNT activity has been reported in radio-resistant Esophageal squamous cell carcinoma (ESCC) cell lines (Zhao et al., 2018), which upon inhibition reversed the radio-resistance phenotype of these cells (Zhao et al., 2018). Mechanistically it was shown that the Wnt signaling upregulated HMGB-1, which is chromatin-associated protein involved in the DNA repair (Zhao et al., 2018). In another study, it was shown that Wnt signaling enhances non-homologous end-joining repair in colorectal cancer, which is mediated by LIG4, a DNA ligase, transactivated by β-catenin (Jun et al., 2016). Together, these reports suggest that Wnt signaling can directly regulate the components of DNA repair machinery, thus affecting the response to the radiation. Moreover, the radiation-induced upregulation of Wnt/β-catenin pathways in squamous cell carcinoma of the head and neck (SCCHN) was shown to decrease the sensitivity of SCCHN to irradiation both *in vitro* and *in vivo* (Jing et al., 2019). Thus, targeting Wnt signaling for overcoming radio-resistance appears to be an attractive approach for cancer treatment.

## Notch, Hedgehog, and Wnt Signaling in Resistance to Immunotherapy

**FIGURE 5:**
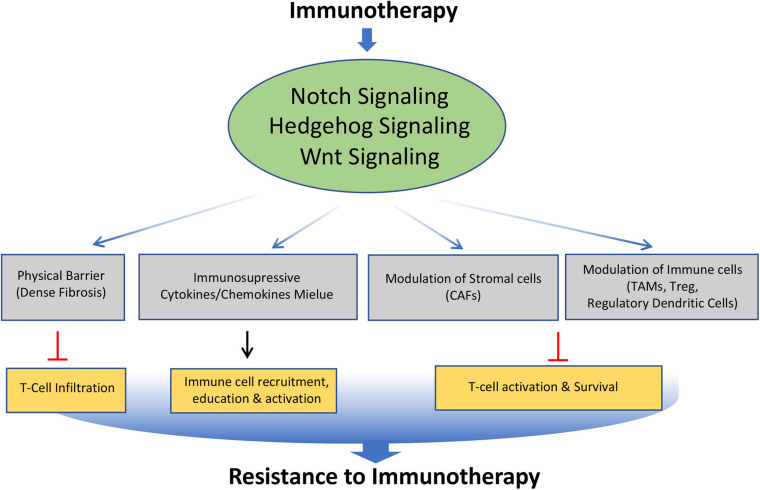
Role of Notch, Hedgehog, and Wnt signaling pathways involved in the acquisition of resistance to immunotherapy. Altered modulation of Notch, Hedgehog, and Wnt signaling by immunotherapy leads to modification of tumor microenvironment such as formation of dense fibrosis, secretion of immunosuppressive cytokines and chemokines, Cancer Associated Fibroblast (CAFs), Tumor Associated Macrophage (TAMs), regulatory dendritic cells. This affects the trafficking, survival and activation of T cells, leading to acquisition of immunotherapy resistance.

Cancer immunotherapy has recently emerged as a new and promising option for the treatment of many malignancies. However, despite the recent successes of cancer immunotherapies, most patients are still refractory with tumors demonstrating resistance to this therapy. Notch, Hedgehog, and Wnt signaling pathways are well known to play an important role in the development, homeostasis, and function of various immune cells. Although limited studies have been carried out, available evidence suggests that these signaling pathways can mediate cancer immune evasion and resistance to immunotherapies through various mechanisms (Figure 5).

### Notch Signaling in Resistance to Immunotherapy

Notch signaling plays multiple roles in the crosstalk between systemic inflammation, myeloid cells in the tumor microenvironment (Franklin et al., 2014), the cancer cell themselves, and multiple lymphocyte subpopulations, thereby modulating tumor immunity (Kuijk et al., 2013). Thus, mutations in the regulators of this pathway are found to be favorable for immunotherapy. In-line with this, melanoma and NSCLC patients who did not respond to immunotherapy showed seldom NOTCH1 mutation, while a marked correlation between NOTCH1/2/3 mutation and better outcome with an immune checkpoint inhibitor (ICI) were found in EGFR/ALK WT NSCLC patients (Zhang et al., 2020). Moreover, the del-NOTCH mutation, which down-regulates Notch signaling, was found to be a potential predictor of favorable ICI response in NSCLC (Zhang et al., 2020). Recent studies have shown that co-occurring mutations in Notch1–3 and homologous recombination repair (HR) genes are associated with increased immunotherapy efficacy in patients with advanced NSCLC (Mazzotta et al., 2020). Moreover, this genomic predictor is also associated with longer survival in patients with other tumor types treated with ICIs (Mazzotta et al., 2020). These observations open the possibility of personalized combination immunotherapy comprising NOTCH inhibitor and ICI in the treatment of NSCLC.

### Hedgehog Signaling in Resistance to Immunotherapy

Hedgehog signaling pathway exerts complex and diverse effects on the tumor immune micro-environment. Emerging evidence suggests that the hedgehog signaling pathway has an immunosuppressive action in many cancers by modulating different components of tumor microenvironments. In Pancreatic ductal adenocarcinoma (PDAC), dense fibrosis is often observed, which acts as a barrier to immune cell infiltration into the tumor, thus making the tumor resistant to immunotherapy. As hedgehog signaling plays an important role in fibrosis, their inhibition has shown a positive effect on immunotherapy. For example, the Hedgehog signaling inhibitor, the Patched 1-interacting peptide has been found to inhibit proliferation and migration of cancer-associated fibroblasts and cancer cells and also increase the infiltration of immune cells by reducing fibrosis of PDAC, thus enhancing the effect of immunotherapy (Oyama et al., 2020). Moreover, Hedgehog (Hh) signaling in myeloid cells is critical for the functioning of Tumor-Associated Macrophages (TAM) M2 polarization and tumor growth (Petty et al., 2019). In this scenario, the sonic HedgehogHedgehog (Shh) secreted by tumor cells drives TAM M2 polarization. The TAM, in turn, suppresses the CD8+ T cells recruitment to the TME, thus mediating the immune-suppression (Petty et al., 2019).

### Wnt Signaling in Resistance to Immunotherapy

Although the role of Wnt signaling in immune cell development remains controversial (Staal et al., 2008; Kabiri et al., 2015), recent studies suggest that it plays an important role in driving the primary, adaptive, and acquired resistance to anticancer immunotherapy (Luke et al., 2019). In human metastatic melanoma samples, an inverse correlation between Wnt/β-catenin pathway activation and T-cell infiltration has been found (Trujillo et al., 2018). Mechanistically, it was shown that the activation of β-catenin signaling in the tumor suppress the expression of chemokines such as CCL4, causing the failure of the dendritic cell recruitment into the tumors, thus resulting in impaired activation of the T cells (Spranger et al., 2015). Moreover, activation of β-catenin in dendritic cells up-regulates the expression of indoleamine 2,3-dioxygenase-1 (IDO) enzyme, which leads to the development of tolerogenic dendritic cells. These transformed dendritic cells favor the differentiation of regulatory T cells, which are involved in the immunotherapy resistance (Holtzhausen et al., 2015).

## Notch, Hedgehog, and Wnt Signaling in Resistance to Targeted Therapy

**FIGURE 6:**
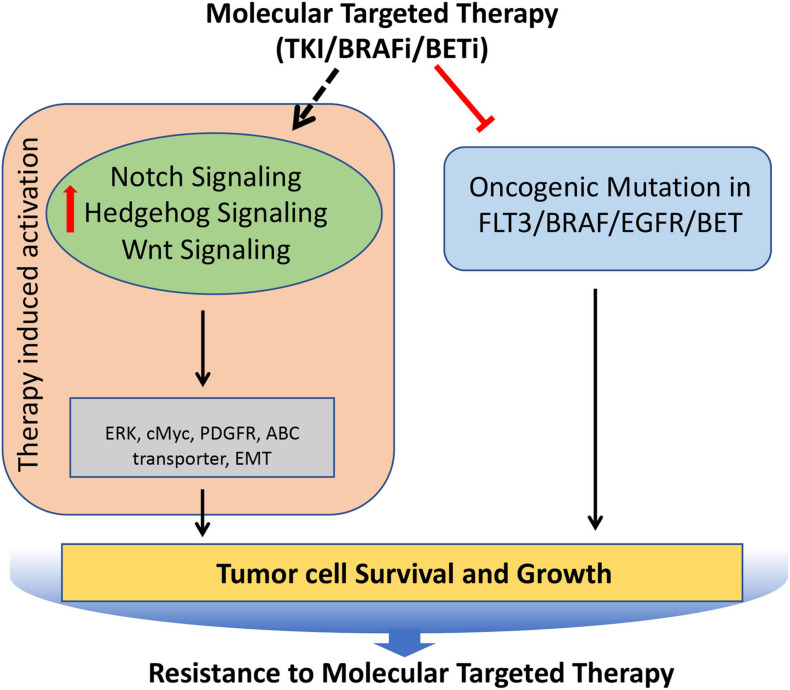
Role of Notch, Hedgehog, and Wnt signaling pathways involved in the acquisition of resistance to molecular targeted therapies in tumors. Molecular targeted therapies block the oncogenic mutated molecular targets in cancer, but often it leads to induced hyperactivation of Notch, Hedgehog, and Wnt signaling pathways, which regulates key molecules (such as cMyc, PDGFR, ERK etc.) involved in the tumor cell survival and growth, resulting in the development of resistance against molecular targeted therapies.

A deep understanding of the critical molecular drivers of cancer has led to the development of targeted therapies that strike the core molecules involved in many malignancies. However, after the initial response, many cancers outsmart such efforts, and thus therapeutic resistance follows, which contributes to cancer mortality. The strategies employed by cancer cells to gain resistance to targeted therapies include activating signals upstream or downstream of oncogenes, direct target reactivation, adaptive survival mechanisms, and engagement of parallel oncogenic pathways. As many of these processes are directly regulated by the Notch, Hedgehog, and Wnt signaling pathways, cancer cells can utilize these signaling pathways to modulate the downstream effectors of the targeted molecules, thereby becoming resistant to the targeted therapies (Figure 6).

### Notch Signaling in Targeted Therapy Resistance

Tumors that acquire resistance to tyrosine kinase inhibitors (TKIs), often show upregulation of the key signaling pathways. For example, genetic alternations of internal tandem duplication (ITD) and mutations of FMS-like tyrosine kinase-3 (FLT3) are most frequently observed in AML. Consequently, FLT3 TKIs are widely used in the treatment of FLT3/ITD^+^ AML patients. Unfortunately, most often, they acquire resistance to TK inhibitors. Mechanistically, Notch signaling was upregulated following treatment with FLT3-TKIs, which resulted in alternative ERK activation. The addition of Notch inhibitor (GSI) abrogated the alternative activation of ERK, resulting in extreme repression of ERK activity, thereby showing a synergistic antitumor effect (Li D. et al., 2020). Likewise, lung adenocarcinoma patients with activating EGFR^L858R^ mutation show a better response to TKIs initially but subsequently develop resistance and show increased HES1 protein levels that correlate with shorter progression-free survival (Bousquet Mur et al., 2020). Administration of Notch inhibitors made these resistant tumors highly responsive to TKIs, thus implicating the role of Notch signaling in providing resistance to the TKIs (Bousquet Mur et al., 2020). BRAF is another molecular target, which is often mutated in many cancer (Davies et al., 2002). Small-molecule BRAF inhibitors (BRAFi) have been developed and have shown a significant survival advantage in patients whose tumors harbor the BRAF driver mutation (Long et al., 2018). In melanomas harboring activating BRAF mutation, the treatment with BRAFi often leads to the development of the resistance to BRAFi. Mechanistically up-regulated Notch signaling is involved in this process, and inhibition of Notch signaling can sensitize these melanomas to BRAF inhibitor (Ruggiero et al., 2019).

### Hedgehog Signaling in Targeted Therapy Resistance

Hedgehog is known to co-operate with the epidermal growth factor receptor (EGFR) signaling pathway during embryogenesis. Therefore, it is not surprising that it is implicated in the resistance against TKIs. The EGFR-TKI resistance NSCLC cells show hyperactivation of hedgehog signaling. This results in EMT induction and ABCG2 up-regulation, and inhibition of hedgehog signaling increases the sensitivity to EGFR-TKIs in resistant NSCLC cells (Bora-Singhal et al., 2015; Della Corte et al., 2015). Moreover, increased expression of Gli1 has been observed in HNSCC cells when subjected to long-term EGFR inhibition. These resistant cells undergo EMT through hedgehog signaling and hedgehog inhibition with Cetuximab delay or completely block tumor recurrence (Keysar et al., 2013). BRAFi induces the activation of the Sonic Hedgehog Homolog (Shh) pathway, which in turn up-regulates the expression of PDGFRα, leading to the resistance of melanoma to BRAFi. Consequently, inhibition of Shh by LDE225 restores and increases the sensitivity of melanoma cells to BRAFi (Sabbatino et al., 2014). Likewise, Gant61 monotherapy reverses the resistance of melanoma cells to Vemurafenib. Interestingly, alternating the dosing schedules of Vemurafenib and Gant61 prevents the onset of BRAFi resistance (Faiao-Flores et al., 2017).

### Wnt Signaling in Targeted Therapy Resistance

Wnt signaling has also been attributed to the development of resistance against targeted therapy. Androgen deprivation therapy (ADT) is the most widely used therapy for the advanced and metastatic prostate cancer (PCa), who cannot be cured by surgery or radiation therapy. Wnt signaling, which is known to be involved in the late stage of PCa, was shown to be activated upon the inhibition of androgen receptor (AR), resulting in therapy resistance to the ADT. Mechanistically, it was shown that the activation of Wnt signaling was not by mutation but through cross talk with other signaling pathways, growth factors, and cytokines produced in response to damaged TME followed by inhibition of androgen receptor (AR) (Yeh et al., 2019). The inhibition of Wnt/β-catenin signaling re-sensitizes the ADT resistant tumors to abiraterone acetate/prednisone (AA/P). Moreover, the reports from the preclinical studies suggest that the combination of an antiandrogen agent with a Wnt pathway inhibitor achieved enhanced growth suppression in prostate cancer (Wang et al., 2008). Wnt signaling has also been shown to be the key player in mediating the resistance to the Bromodomain and extra terminal protein (BET) inhibitors. The BET inhibitors are used as a first-in-class therapy for the treatment of acute lymphoblastic leukemia, but often the patient develops resistance to BET inhibitors. Two independent studies found that the Wnt signaling was involved in the development of primary and acquired resistance to BET inhibitors in acute lymphoblastic leukemia (Fong et al., 2015; Rathert et al., 2015). Mechanistically it was shown that BET inhibitors repress BRD4-dependant expression of MYC oncogene; however, the β-catenin maintains the expression levels of MYC in the presence of BET inhibitors, thereby mediating the resistance to BET inhibitors. Similarly, the role of Wnt signaling in promoting the resistance to BRAF inhibitors and EGFR inhibitors in colorectal cancer and lung cancer has been well documented (Scarborough et al., 2017; Chen et al., 2018).

## Therapeutic Targeting of Notch, Hedgehog, and Wnt Signaling Pathways: Clinical Update and Recent Development of the Inhibitors

Inhibitors of gamma-secretase were the first to enter clinical trials and has been evaluated extensively (Cook et al., 2018). Their merit includes pan-Notch inhibitory activity, favorable tissue distribution, oral administration, and low cost (Andersson and Lendahl, 2014; Kongkavitoon et al., 2018). However, they show gastrointestinal tract toxicity, prompting cautious usage, and restraining further development for clinical applications (Takebe et al., 2014). Anti-DLL4 and DLL3 ligand inhibitors, as well as other pan Notch signaling inhibitors, have also been evaluated in clinics (Cook et al., 2018). As a monotherapy, all Notch inhibitors have been so far assessed for safety and toxicity as well as for dose optimization in Phase I/II trials, which have shown satisfactory patient compliance and toxicity (Du et al., 2019; Yang et al., 2020). Drugs targeting Notch signaling (including antibody against various components of Notch signaling) have also been evaluated as a part of combinational strategies with the standard of care therapies (like chemo- and radiotherapy) in various primary and metastatic tumors (Table 2). Phase I clinical trials have not indicated any additional toxicities induced by combining Notch inhibitors with a different standard of care chemotherapeutic drugs like gemcitabine, cisplatin/carboplatin, folate antimetabolites, taxol, temozolomide, etc. (Du et al., 2019). However, cardiotoxicity has been observed with a combination of antibody-based Notch inhibitors and carboplatin (Du et al., 2019). Phase II clinical trials with gamma-secretase inhibitor RO4929097 in metastatic melanoma and metastatic colorectal cancer have not shown a compelling clinical benefit and, therefore, has not been pursued further (Strosberg et al., 2012; Lee et al., 2015). A correlation between Notch status and response to standard of care therapies has also been not very conclusive. For example, hyperactivation of Notch1 showed no correlation with the response to a combination of methotrexate and cyclophosphamide in children with T-cell acute lymphoblastic leukemia (Clappier et al., 2010). Therefore, there is a need for identifying (or designing) drugs and/or therapeutic strategies with encouraging efficacy and acceptable toxicity, besides establishing a compelling role for targeting Notch signaling in the clinical setting.

**TABLE 2:** Overview of the clinical trials evaluating drugs targeting developmental signaling pathways.

Pathway	Neoplasms included	Targets	Mono-therapy	Combined therapies	Trial stage	Outcome
Notch	Solid tumors; Leukemia	Notch ligand DLL3, DLL4; γ secretase; Pan notch signaling	+	+	I–II	Well tolerated with acceptable toxicity and moderate efficacy
Wnt	Solid tumors; Leukemia	Porcupine; β-catenin/CBP; Frizzled receptor; Fzd8-Fc fusion protein	+	+	I–II	Tolerated with minimal toxicity, but poor efficacy
Hedgehog	Solid tumors; Leukemia	Smoothened	+	+	I–II	Tolerated, but not with encouraging clinical benefit

Drugs targeting Wnt signaling that has been evaluated in clinics include porcupine inhibitors, agents targeting β-catenin/CBP, and Frizzled receptors (Table 2). Drugs targeting smoothed receptors (Smo inhibitors) viz. vismodegib, sonidegib, and glasdegib are among the therapeutics targeting hedgehog signaling, which have been so far evaluated in Phase I/II trials (Table 2). These studies have shown that, unfortunately, tumors develop resistance against Smo inhibitors and are also associated with a variety of toxicities, which limit their clinical evaluation further for efficacy (Du et al., 2019). These inhibitors have also been evaluated in combination with other chemotherapeutic drugs like cisplatin and temozolomide in the treatment of solid tumors with limited benefit (Xie et al., 2019). Arsenic trioxide is an inhibitor of Gli transcription factor and Hedgehog signaling that has also been evaluated in Phase II clinical trials for its efficacy in few solid tumors and hematological malignancies (Xie et al., 2019).

Although clinical trials of drugs to target the three developmental signaling pathways associated with the CSCs was initiated more than a decade back, it has not translated into clinical benefit both due to their inability to reduce the tumor recurrence linked to the CSCs as well as off-target toxicity (Du et al., 2019; Katoh and Katoh, 2020; Yang et al., 2020). Therefore, approaches with the potential to address both these issues have received attention in the recent past. One such approach investigated is the novel nano-drug delivery systems (NDDS) due to their efficient and targeted delivery to the CSC niche in the TME, thereby enhancing the effects on CSC and reducing the off-target effects (Du et al., 2019). For example, Notch inhibitors like DAPT, MRK-560, MK-0752, BMS-906024 with Nanoparticle (NP) carriers are under clinical development for enhanced systemic tumor delivery and limiting the side effects in breast cancer cells (Mamaeva et al., 2011; Mamaeva et al., 2016). Similarly, inhibitors of Wnt signaling like SFRP-1, Niclosamide (NIC), and cromolyn were also delivered by nanoparticle into the tumor microenvironment that blocks the Wnt5a or FRZD-7 receptors and results in Wnt signaling inhibition in TNBC, ovarian cancer, colon, colorectal cancer, and metastatic melanoma (Ghoshal et al., 2015; Liu et al., 2018). The other approach is the use of phytomedicines such as curcumin, epigallocatechin-3-gallate (EGCG), resveratrol, sulforaphane, and genistein, either as monotherapy as in combination with standard of care therapeutics. Interestingly these phytochemicals/phytomedicines show effects on the three developmental signaling pathways, which could contribute to their overall effects, besides other well-established mechanisms of action of these phytochemicals (Yang et al., 2020). Reduced toxicity associated with these phytomedicines is an added advantage that has encouraged the initiation of the Phase I/II clinical trials, which are currently in progress (Table 3; Yang et al., 2020).

**TABLE 3:** Overview of the currently ongoing Phase I/II clinical trials using phytomedicines targeting developmental signaling pathways in solid tumors.

Phytomedicine	Signaling pathway affected			Outcome
	Notch	Wnt	Hedgehog	
Curcumin	+	++	++	Awaited
EGCG	+	++	+	Awaited
Resveratrol	−	+	−	Awaited
Sulforaphane	++	++	+	Awaited
Genistein	−	−	+++	Awaited

## Conclusion

Resistance to anticancer therapy stems from complex and multifactorial processes and has still remained the main reason behind the failure of various therapies. Evolutionary conserved developmental signaling pathways such as Notch, Hedgehog, and Wnt signaling are well recognized for their role in regulating many cellular functions that play key roles in tumor development and progression. These signaling pathways are often upregulated in tumors, which often hijack these pathways to evolve continuously under the pressure induced by the therapy, thereby enabling them to become resistant to various therapies. Many inhibitors developed to target these signaling pathways have shown promising efficacy in preclinical cancer models, and some have even advanced to clinical trials with modest efficacy seen so far. However, as these signaling pathways are broadly active in normal tissue also, targeting these pathways with pan inhibitor often leads to undesirable off-target effects, particularly in the gastrointestinal tract. Therefore, one of the major challenges in the future will be to develop more selective inhibitors that will not only be more effective on tumors but have minimal normal tissue toxicity. Efforts are also required in developing synergistic drug combinations so that lower dosages can be used to improve the therapeutic index.

## Author Contributions

VK and BSD conceptualized the manuscript. VK, MV, and BSD wrote the manuscript. VK and MV prepared the figures. LK, XW, CG, and JL reviewed the draft of the manuscript. All authors read and approved the final manuscript.

## Conflict of Interest

The authors declare that the research was conducted in the absence of any commercial or financial relationships that could be construed as a potential conflict of interest.
